# 1-Linoleoylglycerophosphocholine stimulates UCP1-dependent thermogenesis and mitochondrial respiration to combat obesity

**DOI:** 10.1016/j.jlr.2025.100914

**Published:** 2025-09-26

**Authors:** Rui Wang, Tianfu Zhu, Jingxian Lu, Mengke Cheng, Xingyun Wang, Xirong Guo, Shan Huang, Jianfang Gao

**Affiliations:** 1 Hongqiao International Institute of Medicine, Tongren Hospital, Shanghai Jiao Tong University School of Medicine, Shanghai, China; 2 Department of Pediatrics, Yixing People’s Hospital, Yixing, China; 3 Department of Endocrinology, Tongren Hospital, Shanghai Jiao Tong University School of Medicine, Shanghai, China

**Keywords:** obesity, 1-linoleoylglycerophosphocholine, uncoupling protein 1, mitochondrial respiration, NRF2, KEAP1

## Abstract

Obesity leads to numerous illnesses and metabolic disorders, with lysophosphatidylcholine levels declining in obese patients. However, the physiological role of lysophosphatidylcholine and the regulatory mechanisms involved in modulating obesity remain largely unknown. Here, we provide evidence that 1-linoleoylglycerophosphocholine (1-LGPC) promotes adipocyte energy expenditure by activating the Kelch-like ECH-associated protein 1-nuclear factor erythroid 2-related factor 2 (NRF2) axis. Metabolomic analyses identified 1-LGPC as a characteristic metabolite that declined in the peripheral blood of obese patients. Treatment with 1-LGPC effectively alleviated high-fat diet-induced lipid accumulation in zebrafish larvae and human adipocytes. Elevated expression levels, increased oxygen consumption rates, and enhanced transcript levels indicated that uncoupling protein 1-dependent thermogenesis and mitochondrial respiration were significantly boosted. Furthermore, NRF2 expression and nuclear translocation were induced by 1-LGPC, and NRF2 inhibition triggered uncoupling protein 1 downregulation and lipid accumulation restoration, confirming the Kelch-like ECH-associated protein 1-NRF2 axis’s involvement in 1-LGPC-induced energy expenditure. These findings offer preliminary insights into physiological roles and mechanisms by which 1-LGPC modulates lipid and energy metabolism, providing potential strategies for obesity intervention using clinically identified compounds.

## Graphical abstract

**Figure:**
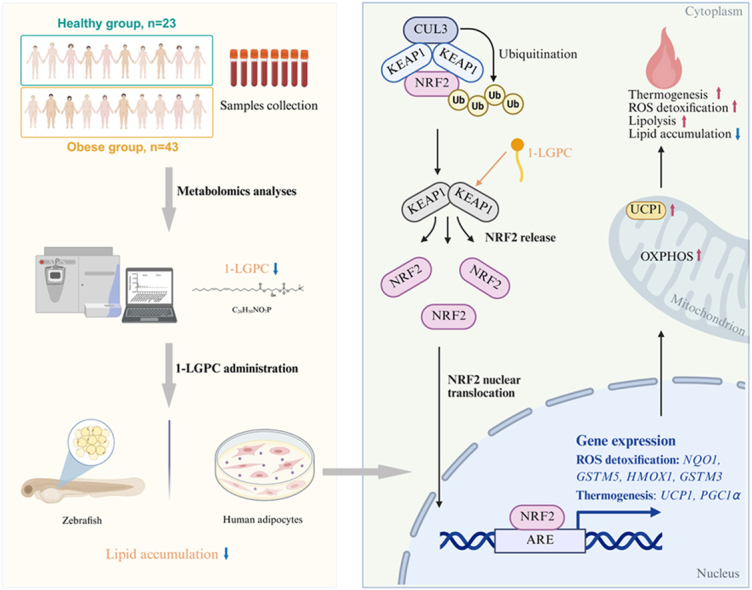

Since 1975, the number of overweight adults worldwide has nearly tripled, reaching over 1.9 billion, with more than 650 million classified as obese (39% overweight and 13% obese), making obesity a true noninfectious pandemic (1, 2). Obesity is a complex illness resulting from a persistent positive energy balance, where energy intake from food surpasses energy expenditure. Excess energy is converted into triacylglycerol (TG), which is stored in expanding adipose tissue depots, increasing body fat and weight gain (3). This accumulation of excess body fat causes a range of metabolic disorders and illnesses, including insulin resistance, nonalcoholic fatty liver disease, prediabetes, type 2 diabetes, and atherogenic dyslipidemia (4). Therefore, effective strategies are urgently needed to combat obesity.

Thermogenic adipocytes, such as those found in brown and beige adipose tissue, possess an extraordinary capacity to oxidize fuels in futile cycles, making them a key focus in obesity treatment (5). Uncoupling protein 1 (UCP1) is highly expressed in brown and beige adipocytes, facilitating nonshivering thermogenesis in response to stimuli like cold, exercise, and food intake. This process increases energy expenditure and reduces the risk of obesity. Additionally, energy expenditure can be elevated without UCP1 through substrate cycling involving oxidized lipids, cold-induced *N*-acyl amino acids, and creatine metabolites in white adipocytes (6). Several small molecules have been reported to reduce lipid accumulation through UCP1-dependent and UCP1-independent thermogenesis (7), including natural compounds (8), endogenous small molecules (9), and synthetic compounds (10). Despite these advancements, there remains a need for more effective molecules and a deeper understanding of the mechanisms involved to treat obesity better.

Lysophosphatidylcholine (LPC), also known as hydrolyzed phosphatidylcholine (PC), can contain either a saturated or an unsaturated fatty acid in the *sn*-1 or *sn*-2 position. LPC is the most abundant lysoglycerophospholipid in human blood, with plasma levels ranging from 150 to 500 μM in healthy individuals (11). The concentrations and roles of LPC vary across different diseases; schistosomal-derived LPC 16:0 activated M2 polarization of macrophages through a PPAR-γ-dependent mechanism (12), and LPC 16:0 increased the release of interleukin-6 and activated SREBP-2 by enhancing cholesterol efflux (13). Additionally, LPC 18:0 protects against endotoxemia and experimental sepsis by inhibiting lipopolysaccharide-mediated caspase-11 activation (14), and LPC 16:0 and LPC 20:4 increased the transcription and protein levels of cyclooxygenase-2 (COX-2) to maintain basal endothelial COX-2 expression, which led to an excess of proinflammatory and vasoconstricting prostanoids in hyperlipidemia (15). Most studies have revealed negative relationships between BMI and various LPC species, particularly for LPC 15:0, LPC 16:0, LPC 17:0, LPC 18:0, LPC 18:1, and LPC 18:2 (16). Regarding physiological functions, LPC 17:0 significantly decreased body weight and improved adipose steatosis in high-fat diet (HFD) mice by stimulating G-protein-coupled receptors and promoting glucagon-like peptide-1 expression (17). It was reported that peripheral LPC could be converted to lysophosphatidic acid, leading to higher cortical excitability and promoting food intake (18). However, the physiological role and regulatory mechanisms of LPC species in obesity remained largely unknown.

Recently, nuclear factor erythroid 2-related factor 2 (NRF2) has garnered significant attention due to its role in upregulating major cytoprotective enzymes in obesity and its related comorbidities (19). NRF2 is a crucial basic leucine zipper transcription factor that is maintained at low basal levels in the cytoplasm under normal conditions by binding to Kelch-like ECH-associated protein 1 (KEAP1), an adaptor protein for cullin3-based ubiquitin E3 ligase. Exposure to electrophiles and reactive oxygen species (ROS) modifies KEAP1 cysteine residues, resulting in the release of NRF2. This release leads to the accumulation and subsequent translocation of NRF2 to the nucleus, where it activates the transcription of numerous ROS-detoxifying enzymes and components of the antioxidant system (20). Obesity is closely associated with inflammation and oxidative stress, and NRF2 has been identified as a key regulator in the browning of white adipose tissue (21). By activating NRF2-KEAP1 signaling, punicalagin has been shown to reduce obesity and the associated inflammatory response (22). Similarly, *Heterotrigona itama* bee bread showed therapeutic potential against oxidative stress and improved lipid metabolism in the liver of obese mice, presumably by activating the NRF2-KEAP1 pathway (23). Nevertheless, more detailed investigations are needed to elucidate the role of the KEAP1-NRF2 axis in obesity.

In this study, we hypothesized that 1-linoleoylglycerophosphocholine (1-LGPC), an LPC species diminished in obese individuals, attenuates lipid accumulation through KEAP1-NRF2 axis activation and mitochondrial thermogenesis. To test this hypothesis, we aimed to validate the antiobesogenic effects of 1-LGPC using zebrafish larvae and human adipocyte models. Furthermore, we will elucidate its mechanism of action on mitochondrial respiration, thermogenesis, and redox homeostasis and determine the necessity of NRF2 signaling in mediating 1-LGPC’s metabolic benefits by specific inhibitor. Collectively, this study will reveal the regulatory mechanism that 1-LGPC activates the KEAP1-NRF2 axis to induce UCP1-dependent thermogenesis, offering a lipid-based therapeutic strategy to combat obesity.

## Materials and Methods

### Participants and clinical measurements

All data and biological specimens used in this study were obtained from the Department of Endocrinology, Shanghai Jiao Tong University School of Medicine Affiliated Tongren Hospital. Patients with BMI ≥30 kg/m^2^ were diagnosed as obese, whereas those with 25 ≤ BMI <30 kg/m^2^ were classified as overweight, according to the World Health Organization criteria. In this study, patients with a BMI ≥25 kg/m^2^ were defined as obese, whereas those with a BMI <25 kg/m^2^ were defined as the healthy group. A total of 43 obese patients (28 males, 15 females; age 48.23 ± 13.22; BMI 29.71 ± 3.60) were recruited, and 23 metabolically healthy controls (Ctrls, 15 males, 8 females; age 44.57 ± 11.64; BMI 22.64 ± 1.33) were recruited from the Clinics of Shanghai Tongren Hospital. Participants were eligible if they were aged 18 years or older, had complete data for all study-related variables, and voluntarily provided written informed consent. Individuals were excluded if they had a history of severe cardiovascular or cerebrovascular diseases, hepatic or renal dysfunction, malignancies, were pregnant, or had a BMI below 18.5 kg/m^2^. This clinical study (no.: 2021-059-01) was approved by the Internal Review and Ethics Boards of Tongren Hospital, affiliated with Shanghai Jiao Tong University. Informed consent was obtained from each patient. The study adhered to the Declaration of Helsinki, country-specific requirements, and good clinical practices.

The subjects’ sex, age, region, body weight, height, waist circumference, blood pressure, and BMI were all evaluated. After fasting for 8–12 h, plasma samples were collected from the individuals. Biochemical indexes such as fasting blood sugar (FBS), glycated hemoglobin A1c, alanine aminotransferase, aspartate aminotransferase, total cholesterol, TG, LDL, HDL, and creatinine were measured using an automatic biochemical analyzer (AU5800 clinical chemistry analyzer; Beckman Coulter, Inc, Brea, CA). Serum insulin and c-peptide were assessed using radioimmunoassay.

### LC-MS-based untargeted metabolomics

Fasting serum samples from 66 individuals (23 Ctrls and 43 newly diagnosed obese cases) were immediately stored at −80°C after assessing clinical indexes. For metabolomic analysis, 300 μl of methanol containing a standard of 5 g/ml L-2-chlorophenylalanine was added to 200 μl of serum samples, which were then vortexed for 2 min. After 10 min of centrifugation at 13,000 rpm and 4°C, 200 μl of the supernatant was recovered. To prepare quality Ctrl samples, equal amounts of serum from each sample were combined and mixed uniformly.

An ultra-performance liquid chromatography (UPLC) system equipped with an electrospray ionization source was utilized for untargeted metabolomics profiling of serum samples. The system scanned an *m/z* range of 100 to 1,500. Data-dependent MS/MS mode was employed, selecting the top 10 most intense ions (Top N = 10) for fragmentation. Full scan MS was performed with a resolution of 17,000 at *m/z* 200, ensuring high accuracy and sensitivity in metabolite detection. Chromatographic conditions were as follows: the injection volume was 2 μl, the column temperature was maintained at 25°C, and the flow rate was set to 0.35 ml/min. The mobile phase consisted of liquid A (an aqueous solution containing 0.1% formic acid) and liquid B (acetonitrile containing 0.1% formic acid). The chromatographic gradient was programmed as: 0–2 min, 5% in liquid B; 2–10 min, 5–95% in liquid B; 10–15 min, 95% in liquid B; and 15–18 min, 5% in liquid B. Data acquisition was performed using Thermo Scientific’s Xcalibur 2.2 software (Thermo Scientific, San Jose, CA).

Peak extraction and alignment were conducted using Compound Discoverer software (Thermo Fisher Scientific), obtaining retention time, *m/z*, and peak area information. Metabolites were identified by matching their exact mass, fragment ion mass, and retention time to a standard metabolite library. An unsupervised principal component analysis (PCA) was performed with Simca-P software, version 13.0 (Umetrics, Umea, Sweden) to assess the general segregation patterns among the samples.

### Multiple reaction monitoring transition

For multiple reaction monitoring (MRM) transition, 300 μl of peripheral blood sample was mixed with 300 μl methanol and 1 ml chloroform via vortex oscillation for 1 min, followed by an addition of 300 μl deionized water and repeated vortexing for 1 min. The mixtures underwent ultrasonication at 4°C for 30 min, static incubation at 4°C for 1 h, and centrifugation at 4°C for 15 min in a TGL-16 high-speed refrigerator. Supernatants were dried using a rotary evaporator, reconstituted in 200 μl methanol, vortexed for 1 min, filtered through 0.22 μm membranes, and diluted 50-fold with methanol prior to LC-MS/MS analysis. Chromatographic separation was performed on a UPLC system equipped with an Acquity UPLC HSS T3 column (1.8 μm, 2.1 mm × 100 mm) maintained at 40°C. The mobile phase consisted of A: water/acetonitrile (6:4, v/v, 5 mM ammonium formate) and B: acetonitrile/isopropanol (1:9, v/v, 5 mM ammonium formate) delivered at 0.300 ml/min under gradient conditions: initial 10% B (0–1 min), linear increase to 90% B (1–2 min), hold at 90% B (2–3 min), return to 10% B (3–4.1 min), and re-equilibration (4.1–5 min). Mass spectrometric detection employed an AB SCIEX 5500 Qtrap-MS with a positive electrospray ionization source under optimized parameters: ion spray voltage 4,500 V, source temperature 450°C, curtain gas 35 arb, collision gas 9 arb, ion source gas 1 55 arb, and ion source gas 2 55 arb. Analyses used the MRM transition *m/z* 520/184 with declustering potential 98 V, collision energy 22 eV, and cell exit potential 11 V, corresponding to the 3.61 min retention window of 1-LGPC (MedChemExpress).

### Zebrafish rearing and treatment with 1-LGPC

The *Tu* strain zebrafish larvae used in this study were provided by the China Zebrafish Resource Center. Healthy fertilized zebrafish eggs, collected at 24 h postfertilization, were placed in E3 fish water containing 5 mM NaCl, 0.17 mM KCl, 0.33 mM CaCl_2_, 0.33 mM MgSO_4_, 0.1 μg/ml methylene blue, and 200 μM 1-phenyl 2-thiourea to inhibit melanin buildup. Six hours after fertilization, healthy fertilized eggs were harvested and placed in a 6-well cell culture plate. Larvae were randomized into various treatment groups before feeding, with 17 zebrafish larvae in each treatment group. They were fed either a Ctrl diet (paramecium solution) or an HFD (dilution of 1:10 clotted cream in E3 fish water with 200 μM 1-phenyl 2-thiourea; Devon Cream Company) for 6 h per day from 4 to 6 days postfertilization (dpf) at 28°C (24). Zebrafish larvae treated with 1-LGPC (50 μM) between 4 and 6 dpf were fed either an HFD or a Ctrl diet for 6 h daily. Lipid staining analysis of the zebrafish was conducted 18 h following the final feeding day. The animal experiments were approved by the Tongren Hospital’s Animal Ethics Committee, China (no.: A2022-016-01, dated: October 27, 2022).

### Zebrafish larvae lipid staining

Zebrafish larvae at 7 dpf were fixed in 4% paraformaldehyde overnight at 4°C. After three 5-min washes with PBS, the zebrafish larvae were incubated in 60% isopropanol for 30 min. They were then stained with freshly prepared Oil Red O staining solution (ScienCell) for 3 h. Following three additional 5-min washes in 60% isopropanol, the larvae were transferred to PBS. The treated larvae were then examined using a stereomicroscope (Zeiss, Germany). For Nile Red staining, zebrafish larvae were collected and incubated in 5 ml of E3 fish water containing 0.5 μg/ml Nile Red solution (prepared from a 1.25 mg/ml stock solution in acetone, diluted in E3 fish water) for 30 min at room temperature in the dark. After incubation, the zebrafish were imaged using a Zeiss LSM880 confocal microscopy (Zeiss, Germany).

### Culture and differentiation of human preadipocytes

The human preadipocytes obtained from ScienCell Research Laboratories were cultivated in PAM medium (ScienCell) with 10% fetal bovine serum at 37°C and 5% CO_2_. Five generations were involved in the cell passing. Three days after confluency, the culture medium was changed to a differentiation medium. A cocktail of DMEM/F12, 0.5 mM 3-isobutyl-1-methylxanthine, 0.5 μM insulin, 1 μM dexamethasone, and 1 μM rosiglitazone (all from Sigma) was used to start the differentiation. Following 4 days of induction, cells were moved to a maintenance medium that included DMEM/F12 and 0.5 μM insulin for 4 days. Every 2 days, the culture media were replaced. To analyze the adipocyte differentiation that 1-LGPC caused, cells were treated with 1-LGPC during differentiation (MCE; percent purity is HPLC ≥99.85%). 1-LGPC was dissolved in absolute methanol for the studies, and the Ctrl cells were treated with the same volume solution. By day 8, the efficacy of adipocyte differentiation was evaluated using Oil Red O staining. In the NRF2 inhibition experiment, preadipocyte cultures underwent sequential treatments: initial exposure to the NRF2 signaling suppressor brusatol (200 nM; Selleckchem, S7956) for 30 min, followed by administration of 1-LGPC for 48 h.

### Cell viability assessment and Oil Red O staining

The impact of 1-LGPC on human preadipocyte proliferation was assessed using the Cell Counting Kit 8 (CCK8) assay (Beyotime, China). Human preadipocytes were seeded in a 96-well plate at a density of 2000 cells per well in 100 μl of medium. Various concentrations of 1-LGPC (0, 25, 50, 100, and 200 μM) were administered to the cells for 24, 48, and 72 h. At the end of each treatment period, 10 μl of CCK8 solution was added to each well. Following 1 h of incubation in a 5% CO_2_ incubator at 37°C, the absorbance was measured at a wavelength of 450 nm using a Tecan microplate reader. Six replicates were used for each sample in all trials. The cell survival rate was calculated using the formula: [(*As* - *Ab*)/(*Ac* - *Ab*)] × 100%, where *As* is the absorbance of the experimental well (containing cell, culture medium, CCK8 solution, and 1-LGPC), *Ac* is the absorbance of Ctrl well (containing cells, culture medium, CCK8 solution, but no 1-LGPC), and *Ab* is the absorbance of the blank well (containing culture medium and CCK8 solution, but no cells and no 1-LGPC).

Following a PBS wash, the treated adipocytes were fixed in 4% paraformaldehyde for 20 min at room temperature. The cells were then stained with a 3:2 solution of Oil Red O solution and water for 10 min. After staining, the cells were washed four times with distilled water and examined under a light microscope.

### Gene expression analysis

Total RNA was isolated from adipocytes using the RNA-easy™ isolation reagent (Vazyme, China) and quantified using a Nanodrop 2000 UV-visible spectrophotometer. Using 1 μg of RNA, complementary DNA was synthesized with PrimeScript™ RT Master Mix reagent kit (Takara, Japan) following the manufacturer’s directions. Quantitative PCR analyses were performed with primers listed in Table 1, using PowerUp™ SYBR™ Green Master Mix (Applied Biosystems) on a 7900 HT Fast Real-Time PCR System (Applied Biosystems). The PCR amplification protocol included an initial denaturation at 95°C for 1 min, followed by 40 cycles of 15 s at 95°C, 15 s at 60°C, and 1 min at 72°C. Relative gene expression levels were calculated using the 2^−ΔΔCt^ method, with *PPIA* RNA expression serving as an internal reference for normalization. Each measurement was performed in triplicate.

**Table 1:** Sequences of quantitative PCR primers

Gene	Forward sequence (5′-3′)	Reverse sequence (5′-3′)
*PPIA*	TTCATCTGCACTGCCAAGAC	TCGAGTTGTCCACAGTCAGC
*UCP1*	CTGGAATAGCGGCGTGCTT	AATAACACTGGACGTCGGGC
*PGC1α*	CTGTGTCACCACCCAAATCCTTAT	TGTGTCGAGAAAAGGACCTTGA
*PPARγ*	GCTGTGCAGGAGATCACAGA	GGGCTCCATAAAGTCACCAA
*PPARα*	GGCGAACGATTCGACTCAAG	TCCAAAACGAATCGCGTTGT
*CEBPα*	CTGTGTCACCACCCAAATCCTTAT	TGTGTCGAGAAAAGGACCTTGA
*PLIN1*	CACCTGCCTTACATGGCTTG	TTCTGGAAGCATTCGCAGGT
*ATGL*	GTGTCAGACGGCGAGAATG	TGGAGGGAGGGAGGGATG
*HSL*	GGCACTTTCTGAGTGGGTCA	CGACGTCCCACTGTATCCTG
*FASN*	TGAACTCCTTGGCGGAAGAGA	GTAGGACCCCGTGGAATGTCA
*ACC*	ATGTCTGGCTTGCACCTAGTA	CCCCAAAGCGAGTAACAAATTCT

### Western blot analysis

Cells were lysed in RIPA buffer (Beyotime, China) containing a protease and phosphatase inhibitor cocktail. Protein levels were determined using a BCA Protein Assay Kit (Beyotime, China). The proteins were separated using 12% SDS-PAGE and then transferred to PVDF membranes (Millipore). The membranes were blocked in QuickBlock™ blocking buffer (Beyotime, China) for 15 min at room temperature. Following blocking, the membranes were incubated overnight at 4°C with the following primary antibodies: PPARγ (Abcam, ab272718, 1:10,00 dilution), ATGL (Proteintech, 55190-1-AP, 1:1,000 dilution), hormone-sensitive lipase (HSL) (Proteintech, 17333-1-AP, 1:5,000 dilution), phosphorylated HSL (p-HSL) (CST, 45804, 1:1,000 dilution), PPAR coactivator-1α (PGC1α; Proteintech, 66369-1-Ig, 1:10,000 dilution), UCP1 (CST, 72298S, 1:1,000 dilution), KEAP1 (Beyotime, AF7335, 1:2,000 dilution), NRF2 (Abcam, ab62352, 1:20,000 dilution), and β-actin (Affinity Biosciences, AF7018, 1:1,000 dilution). The membranes were then incubated for 2 h with horseradish peroxidase-conjugated secondary antibodies (GeneTex, GTX213111-01, 1:1,000 dilution). After washing with Tris-buffered saline with Tween-20, protein bands were visualized using ECL (Tanon, China) and quantified using ImageJ 1.51j8 (National Institutes of Health, Bethesda, MD). Each blot represents an average of three or more separate experiments. To account for variations in protein amount and quality, results were normalized to β-actin expression.

### MitoTracker staining

Following treatment with 1-LGPC or Ctrl, a 100 nM working solution of MitoTracker Red CMXRos (Beyotime, China) was prepared for staining. The treated human adipocytes were incubated with the MitoTracker Red dye for 30 min at 37°C. After incubation, the cells were washed three times with preheated PBS. Subsequently, the nuclei were stained with 4',6-diamidino-2-phenylindole (DAPI) (Beyotime, China) for 10 min at 25°C. Finally, the cells were examined using a fluorescent microscope. The MitoTracker Red signal was excited at 561 nm, and the fluorescence was recorded at 600 nm. The fluorescence of DAPI was measured by excitation at 364 nm and recorded at 454 nm.

### Seahorse assay

The Seahorse assay was performed using a Seahorse XF24 extracellular flux analyzer (Agilent) per the manufacturer’s directions. Preadipocytes were seeded at a density of 4,000 cells/well in a 24-well Seahorse assay plate (Agilent) and allowed to differentiate as described previously. The following wells (A1, B4, C3, and D6) received 300 μl of cell-free media and served as background Ctrls. Adipocytes were then treated with 100 μM 1-LGPC for 24 h, concurrently, an Agilent XFe24 flux assay plate with 1 ml Calibrant solution was equilibrated overnight at 37°C. After treatment, adipocytes were incubated in a prewarmed assay medium (XF base medium with 10 mM glucose, 1 mM sodium pyruvate, and 2 mM glutamine) for 1 h without CO_2_ before oxygen consumption analysis. Oxygen consumption rate (OCR) was then assessed under basal conditions and after the sequential injection of different reagents (103015-100; Agilent), including ATP synthase inhibitor oligomycin A (1.5 μM), carbonyl cyanide 4-(trifluoromethoxy) phenylhydrazone (0.5 μM), and rotenone/antimycin A (0.5 μM). The OCR values were normalized to protein content.

### Immunofluorescence staining

Human preadipocytes were permeabilized with 1% Triton X-100 in PBS at 25 °C for 10 min after being fixed with 4% paraformaldehyde for 1 h and then washed three times with PBS. After permeabilization, the samples were blocked with 10% bovine serum albumin in PBS for 1 h at room temperature. Following blocking, the samples were incubated at 4°C with primary antibodies: UCP1 rabbit antibody (CST; 72298S, 1:100 dilution) and NRF2 rabbit antibody (Abcam; ab62352, 1:20,000 dilution). The next day, after three PBS washes, the samples were incubated for 1 h at 37°C with the appropriate secondary antibodies, protected from light. The secondary antibodies used were CoraLite488-conjugated goat anti-rabbit IgG(H+L) (Proteintech; SA00013-2, 1:200 dilution). Finally, the samples were stained with DAPI for 10 min at room temperature to label the nuclei. Fluorescent signals were recorded using a fluorescence microscope.

### RNA sequencing and data analysis

Following a 72-h treatment with 100 μM of 1-LGPC, three samples per group were randomly selected for RNA sequencing analysis. Approximately 1 μg of total RNA was used as the input material for sample preparation. The RNA sequencing was performed by Gene Denovo Biotechnology Co (Guangzhou, China). Differentially expressed genes (DEGs) were identified based on the criteria of |log_2_FC| ≥1 and *p* adjust <0.05. The DEGs were subsequently subjected to gene ontology annotation analysis, Kyoto Encyclopedia of Genes and Genomes enrichment analysis, and gene set enrichment analysis (GSEA).

### Lipid extraction and LC-MS/MS analysis

After mixing and vortexing with 250 μl of water for 60 s, the cell samples were frozen and thawed with liquid nitrogen three times and sonicating it for 20 min in an ice-water bath. The homogenate (50 μl) was taken for the quantification of BCA. The extract solution (800 μl; 480 μl of extraction solution including internal standard; MTBE:MeOH = 5:1) was combined with 200 μl of homogenate. The samples were vortexed for 60 s and then sonicated in an ice-water bath for 10 min. The samples were then centrifuged for 15 min at 4°C and 3,000 rpm. The supernatant (500 μl) was moved to a new tube and dried at 37°C in a vacuum concentrator. The dried samples were then rehydrated in 150 μl of resuspension buffer (DCM:MeOH:H_2_O = 60:30:4.5) followed by 30-s vortex mixing and 10-min ice-water bath sonication. After centrifugation for 15 min at 4 °C and 12,000 rpm, 70 μl of the resulting supernatant was collected into a new glass vial for subsequent LC-MS analysis.

Chromatographic separation was performed using an ACQUITY Premier UHPLC System with dual mobile phases: phase A contained 40% water, 60% acetonitrile, 10 mM ammonium acetate, and 0.1% acetic acid; phase B comprised 10% acetonitrile, 90% isopropanol, 10 mM ammonium acetate, and 0.1% acetic acid. The system operated at 45°C column temperature with a 10°C autosampler and 2 μl injection volume. Mass spectrometric detection was conducted using a SCIEX Triple Quad™ 6500+ instrument configured with the following parameters: ion spray voltage +5,500/-4,500 V, 35 psi curtain gas, 350°C source temperature, dual 50 psi ion source gases, and ±80 V declustering potential. Quantitative analysis was executed through SCIEX Analyst Work Station (version 1.6.3) and DATA DRIVEN FLOW (v2.0.3.11) platforms. Lipid quantification involved calculating absolute concentrations using lipid class-specific internal standards, with final values normalized against protein content measurements.

### CD spectra of proteins

Protein and 1-LGPC samples were solubilized in water and incubated at 37°C for 5 h, maintaining a compound-to-protein final concentration ratio of 4:1. The samples were diluted with water to a concentration of 0.01 μg/μl and measured using the Jasco J-810 spectropolarimeter (Jasco, Tokyo, Japan). The parameters for far-ultraviolet detection were as follows: begin wavelength 190 nm, end wavelength 260 nm, scanning speed 50 nm/min, response 4 s, bandwidth 1 nm, accumulation time 3 times, and cuvette width 1 cm. A blank Ctrl solution with a sample volume of 300 μl was used for baseline correction and subtracted after measurement. The actual sample, also with a volume of 300 μl, was then tested, and the secondary structure was analyzed using CDNN 2.1 (ACGT Progenomics AG, Germany).

### Statistical analysis

All experiments were conducted in three or more biological replicates, and the results were presented as mean ± SD. Statistical analysis was performed using GraphPad Prism 8.0.1 and SPSS 16.0. Student’s *t*-tests were employed to calculate *P* values, with values less than 0.05 considered statistically significant.

## Results

### Levels of the 1-LGPC metabolite are diminished in the peripheral blood of obese patients

To identify differences in metabolite profiles triggered by obesity, 43 obese cases and 23 Ctrls were recruited. The participants in the obesity group ranged in age from 23 to 72 (median, 47; interquartile range, 37–58.5), with men comprising 65% of the group. Age and race/ethnicity did not differ statistically from the Ctrl group (Table 2). In obese patients, total cholesterol, TG, and LDL concentrations were higher than those in the Ctrl group, whereas the HDL concentration was lower. Additionally, FBS, 2-h postprandial plasma glucose, fasting insulin, hemoglobin A1c (3 months), alanine aminotransferase, and aspartate aminotransferase levels were elevated in the obese group, along with systolic blood pressure, diastolic blood pressure, and heart rate. BMI and waist circumference were also significantly increased in obese cases. The participants’ detailed baseline characteristics for the demographic and clinical analysis are listed in Table 2.

**Table 2:** Baseline characteristics of healthy Ctrl and obese patients

Name	Ctrl group, N = 23	Obesity group, N = 43	*P*
Age	44.57 ± 11.64	48.23 ± 13.22	0.268
Sex male, n (%)	15 (65)	28 (65)	0.994
TC	3.84 ± 0.76	4.84 ± 1.15	<0.001
TG	1.09 ± 0.37	2.01 ± 0.91	<0.001
HDL	1.07 ± 0.25	0.94 ± 0.20	0.021
LDL	2.22 ± 0.73	3.41 ± 1.29	<0.001
FBS	5.11 ± 0.46	8.30 ± 3.26	<0.001
Two-hour blood sugar	6.95 ± 0.75	17.32 ± 4.42	<0.001
Fasting insulin	59.07 ± 19.21	102.59 ± 79.23	0.012
HbA1c (3 months)	5.55 ± 0.29	10.04 ± 2.07	<0.001
ALT	22.52 ± 7.88	46.98 ± 28.14	<0.001
AST	23.13 ± 7.85	31.56 ± 14.09	0.010
Creatinine	62.22 ± 9.54	55.82 ± 14.72	0.065
GFR	129.73 ± 18.55	138.69 ± 27.47	0.166
SBP, mm Hg	125.04 ± 9.17	137.67 ± 18.86	0.004
DBP, mm Hg	80.09 ± 6.49	87.14 ± 14.05	0.026
Heart rate	75.78 ± 6.01	87.72 ± 13.65	<0.001
Fasting C-peptide	0.52 ± 0.14	0.56 ± 0.25	0.397
BMI, kg/m^2^	22.64 ± 1.33	29.71 ± 3.60	<0.001
Waist	86.52 ± 6.44	102.40 ± 10.16	<0.001

ALT, alanine aminotransferase; DBP, diastolic blood pressure; GFR, glomerular filtration rate; HbA1c, glycated haemoglobin A1c; SBP, systolic blood pressure; TC, total cholesterol.

**Fig. 1:**
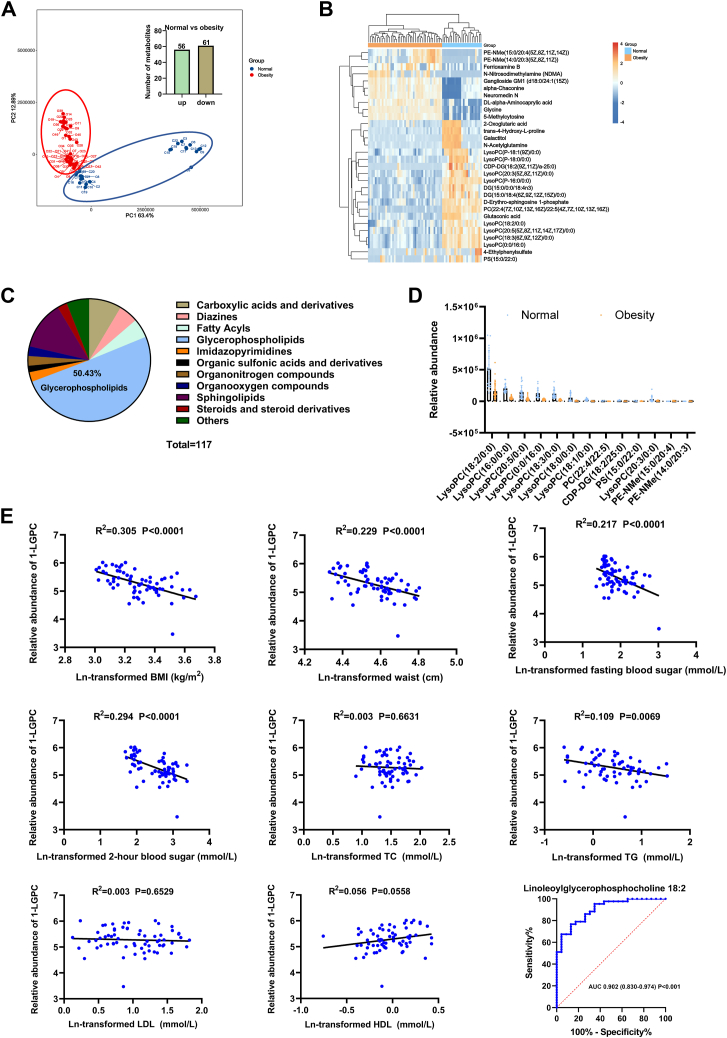
Metabolomic analyses reveal characteristic metabolites in the peripheral blood of obese patients. A: PCA score plot of metabolomic datasets, showing the separation between normal and obese cases and the number of differentially abundant metabolites identified. B: Hierarchical clustering analysis of the top 30 differential metabolites between normal and obese cases. C: Categorization of total differentially abundant identified metabolites. D: Relative abundances of 13 differential glycerophospholipids. E: Partial correlation analysis between the relative abundance of 1-LGPC (lysoPC(18:2/0:0)) and baseline characteristics, along with the receiver operating characteristic curve of 1-LGPC for predicting obesity. CDP-DG, cytidine diphosphate-diacylglycerol; DG, diacylglycerol; PE-NMe, monomethyl phosphatidylethanolamine; PS, phosphatidylserine; TC, total cholesterol.

Peripheral blood samples from cases were collected and analyzed for metabolomic profiles, resulting in the identification of 118 differentially abundant metabolites in the final dataset following data curation and annotation. PCA showed a clear separation between the baseline metabolic profiles of the healthy and obese groups, whereas 76.29% of the variance was explained by the first two principal components, indicating a significant change in metabolism in obese patients compared with Ctrls (Fig. 1A). Compared with the peripheral blood of normal cases, 117 differentially regulated metabolites were identified in obese patients, with 56 metabolites upregulated and 61 downregulated. Among the top 30 differentially regulated metabolites, 66.67% were upregulated (Fig. 1B). Categorization of these metabolites revealed that 50.43% belonged to glycerophospholipids, followed by sphingolipids, carboxylic acids and derivatives, diazines, and fatty acyls (Fig. 1C). Among the glycerophospholipids, 1-LGPC was the most abundant in the top 30 differentially regulated metabolites (Fig. 1D). Therefore, 1-LGPC was selected for further evaluation to assess its relationship with metabolic variations.

Partial correlations between the relative abundance of 1-LGPC and BMI, waist, FBS, 2-h postprandial plasma glucose, and TG showed significant relationships, with *R*-squared values ranging from 0.109 to 0.305 (*P* < 0.01) (Fig. 1E). This suggests that the relative abundance of 1-LGPC in peripheral blood tends to decrease during obesity progression. Sensitivity analysis demonstrated that the correlation between the relative abundance of 1-LGPC and obesity remained robust, with a receiver operating characteristic area under the curve value calculated to be 0.902 (0.830–0.974) (*P* < 0.001). These results indicate that 1-LGPC is a characteristic metabolite that declines in the peripheral blood of obese patients.

### 1-LGPC treatment alleviates lipid accumulation in zebrafish larvae

**Fig. 2:**
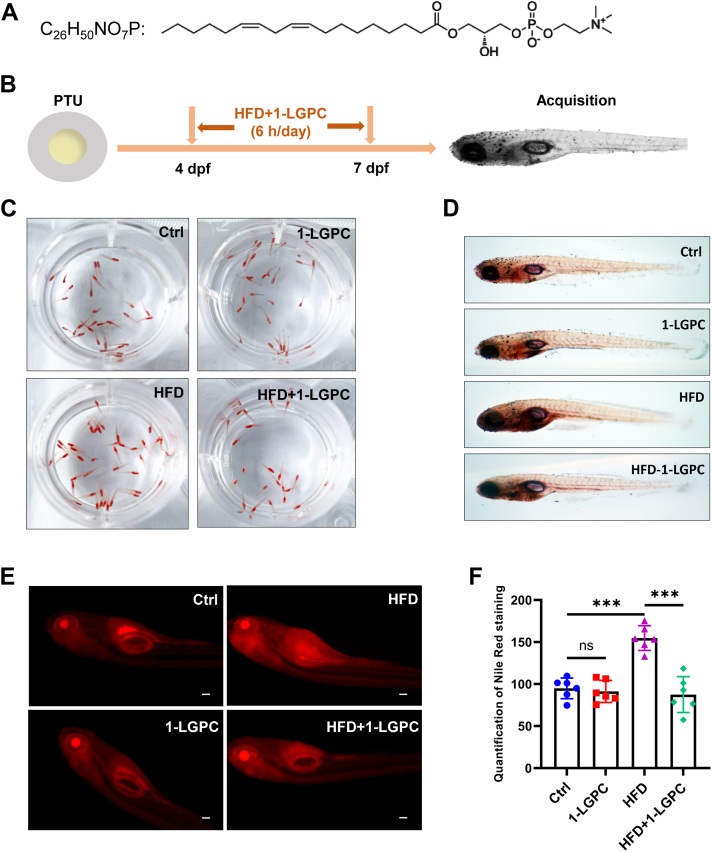
Treatment of 1-LGPC reduces HFD-induced lipid accumulation in zebrafish larvae. A: Molecular formula and structure of 1-LGPC. B: Schematic diagram of the procedure to examine the effect of 1-LGPC against HFD. Zebrafish larvae were fed with a standard diet (Ctrl) or HFD for 6 h every day from 4 to 7 dpf. For 1-LGPC treatment, 50 μM 1-LGPC was added to the zebrafish larvae during either the Ctrl or HFD. C: Representative images of 7 dpf zebrafish larvae fixed and stained with Oil Red O. D: Representative images of 7 dpf zebrafish larvae stained with Oil Red O under the light microscope (4×). E: Representative images of 7 dpf zebrafish larvae stained with Nile Red under the immunofluorescence microscope. The scale bars represent 100 μm. F: Quantification of Nile Red staining in 7 dpf zebrafish larvae with Ctrl or 1-LGPC treatment. Values are mean ± SD (n = 3). Student’s *t*-tests were performed to determine statistically significant differences: ns, no significant; ∗*P* < 0.05; ∗∗*P* < 0.01; and ∗∗∗*P* < 0.001.

The chemical structure of 1-LGPC, which was identified using an MRM approach (supplemental Fig. S1), consists of a glycerol backbone, a hydrophilic polar choline headgroup, and a hydrophobic linoleoyl side chain at *sn*-1 (Fig. 2A). Given the significant deficiency of 1-LGPC in obese patients, its potential effect on obesity was investigated using zebrafish larvae. A diet-induced obese zebrafish model was developed by feeding 4 dpf larvae with high-fat creams to evaluate the benefits of 1-LGPC therapy against HFD-induced obesity. Both normal and diet-induced obese mice were treated with 1-LGPC and given either a standard diet or an HFD diet (Fig. 2B).

Oil Red O staining showed that lipid accumulation was significantly increased by HFD compared with the standard diet (Fig. 2C, D) in Ctrl larvae. Treatment with 1-LGPC reduced lipid accumulation in HFD-fed zebrafish, without affecting the lipid accumulation in normal zebrafish larvae. Furthermore, the ability of 1-LGPC to prevent obesity was assessed using Nile Red staining and quantification (Fig. 2E, F). The relative abundance of Nile Red staining in the HFD-fed zebrafish group was 163.14% of the standard diet group. 1-LGPC treatment reduced Nile Red staining by 43.48% in the HFD-fed zebrafish group while not affecting lipid accumulation in normal zebrafish larvae. Therefore, 1-LGPC effectively alleviated HFD-induced lipid accumulation in zebrafish larvae.

### 1-LGPC attenuates lipid accumulation by enhancing lipolysis and thermogenesis in human adipocytes

**Fig. 3:**
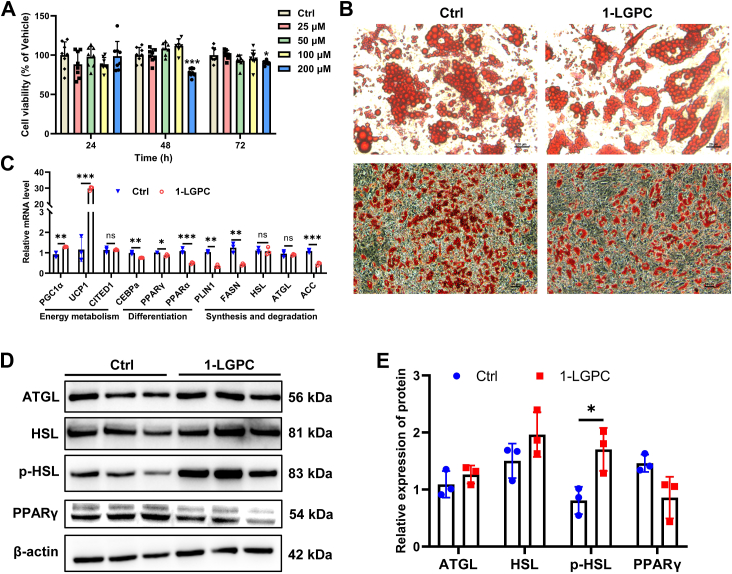
Treatment of 1-LGPC attenuates lipid accumulation in human adipocytes. A: Cell viability of preadipocytes after being exposed to different concentrations of 1-LGPC (0, 25, 50, 100, and 200 μM) for 24, 48, and 72 h. Cell viability was calculated as a percentage relative to preadipocytes exposed to 0 μM of 1-LGPC (Ctrl). B: Representative images of mature adipocytes stained by Oil Red O. The scale bars represent 20 μm (top row); the scale bars represent 50 μm (bottom row). C: Relative mRNA levels of genes involved in energy metabolism, differentiation, and fatty acid synthesis and degradation in the 1-LGPC-treated group compared with Ctrl. D: Immunoblot analysis of FASN, ATGL, HSL, p-HSL, and PPARγ in the 1-LGPC-treated group compared with Ctrl. β-actin was used as an internal reference. E: Relative expression levels of proteins in the 1-LGPC-treated group compared with Ctrl. ACC, acetyl-CoA carboxylase; ATGL, adipose triglyceride lipase; CEBPα, CCAAT-enhancer binding protein α; CITED1, Glu/Asp-rich carboxy-terminal domain 1; *PLIN1*, perilipin 1. Values are mean ± SD (n ≥ 3). Student’s *t*-tests were performed to determine statistically significant differences: ns, no significant; ∗*P* < 0.05; ∗∗*P* < 0.01; and ∗∗∗*P* < 0.001.

The antiobesity effect of 1-LGPC and its associated mechanisms were further investigated in human adipocytes. First, the cytotoxicity of 1-LGPC to preadipocytes was evaluated by exposing them to varying doses of 1-LGPC (0, 25, 50, 100, and 200 μM) for 24, 48, and 72 h. No differences in relative cell viability were observed at concentrations of 25, 50, and 100 μM. However, at a concentration of 200 μM 1-LGPC, a decrease in cell viability by 21.97% was observed at 48 h and 9.86% at 72 h (Fig. 3A). Based on these findings, 100 μM of 1-LGPC was selected for further investigation in human adipocytes.

Next, the effects of 1-LGPC on lipid accumulation were investigated in human adipocytes by inducing adipocyte differentiation and staining with Oil Red O to visualize lipid deposition. Adipocytes treated with 1-LGPC (differentiation day 8) were smaller in volume compared with the Ctrl group, and the amount of lipid droplets was significantly reduced in 1-LGPC-treated adipocytes (Fig. 3B). Meanwhile, the effects of LPC 18:0 and LPC 18:1, two other downregulated LPC species, were also investigated, and the results showed that 100 μM LPC 18:0 and 30 μM LPC 18:1 had no significant effect on lipid accumulation in adipocytes (supplemental Fig. S2). These findings indicated that 1-LGPC specifically diminishes lipid accumulation in human adipocytes.

To explore the underlying mechanism, key genes involved in energy metabolism, differentiation, and fatty acid synthesis and degradation were analyzed. By quantitative PCR, the transcription levels of genes encoding PGC1α and UCP1 were significantly upregulated (Fig. 3C), suggesting enhanced energy metabolism. Conversely, the expression of enzymes involved in cell differentiation, including CCAAT-enhancer binding protein α, PPARγ, and PPARα, was downregulated. Additionally, genes with roles in fatty acid synthesis, including perilipin 1 (*PLIN1*), *FASN*, and acetyl-CoA carboxylase (*ACC*), were also downregulated (Fig. 3C). Although the transcription level of *HSL*, a key enzyme in lipolysis, did not change significantly (Fig. 3C), the level of p-HSL was elevated by 110.86%, as determined by Western blotting (Fig. 3D, E). These results suggest that 1-LGPC attenuated lipid accumulation by enhancing lipolysis and thermogenesis while inhibiting cell differentiation and fatty acid biosynthesis in human adipocytes.

### 1-LGPC enhances mitochondrial respiration and thermogenesis by upregulating PGC1α and UCP1

**Fig. 4:**
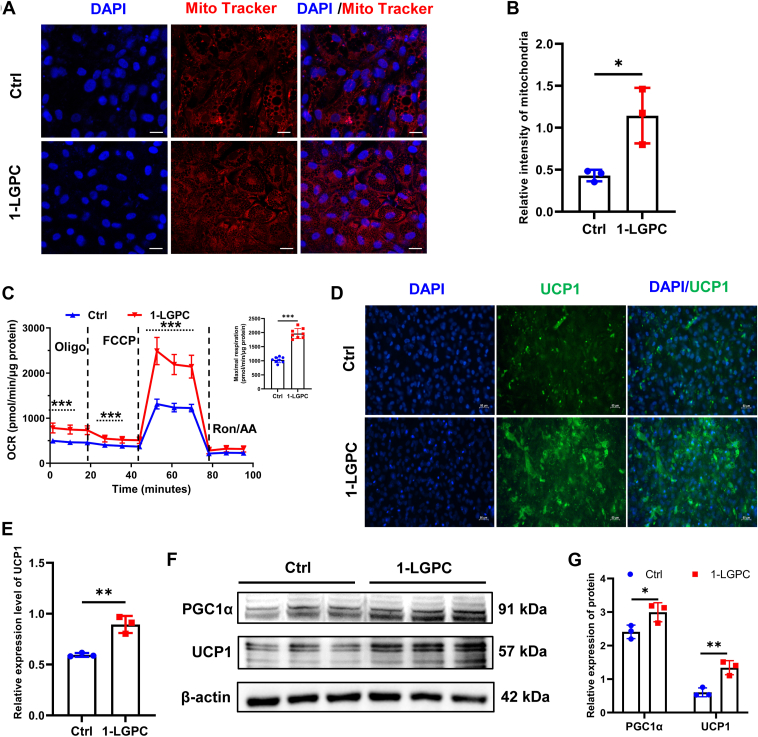
1-LGPC treatment enhances mitochondrial respiration and expression of UCP1. A: Representative images of mitochondria stained with MitoTracker Deep Red. Nuclei were counterstained with DAPI. The scale bars represent 25 μm. B: Relative fluorescence intensity of mitochondria in the 1-LGPC-treated group compared with Ctrl. C: Mitochondrial OCRs and maximal respiration in adipocytes after treatment with 1-LGPC and Ctrl. D: Representative images of UCP1 immunofluorescent staining. Nuclei were counterstained with DAPI. The scale bars represent 50 μm. E: Relative fluorescence intensities of UCP1 in the 1-LGPC-treated group compared with Ctrl. F: Immunoblot analysis of PGC1α and UCP1 in the 1-LGPC-treated group compared with Ctrl. β-actin was used as an internal reference. G: Relative expression levels of proteins in the 1-LGPC-treated group compared with Ctrl. FCCP, carbonyl cyanide p-trifluoromethoxyphenylhydrazone; Oligo, oligomycin; Ron/AA, rotenone/antimycin A. Values are mean ± SD (n ≥ 3). Student’s *t*-tests were performed to determine statistically significant differences: ns, no significant; ∗*P* < 0.05; ∗∗*P* < 0.01; and ∗∗∗*P* < 0.001.

Thermogenic adipocytes enable heat production by coordinating substrate supply with mitochondrial oxidative machinery and effectors in order to modulate the rate of substrate oxidation (25). By MitoTracker Deep Red staining, we observed a 1.65-fold increase in the relative fluorescence intensity as well as a tighter mitochondrial arrangement following 1-LGPC treatment (Fig. 4A, B). The OCR of 1-LGPC-treated cells also remained higher than that of Ctrl group before the addition of oligomycin and after processing with oligomycin and carbonyl cyanide p-trifluoromethoxyphenylhydrazone (Fig. 4C), indicating greater basal respiration (1.80-fold), ATP-linked respiration (2.60-fold), proton leak (1.36-fold), maximal respiration (1.91-fold), and spare respiratory capacity (1.94-fold). These results indicated that 1-LGPC enhances mitochondrial respiration.

UCP1 is critical for the induction of proton leak in brown and beige adipocytes, facilitating the best-characterized mechanism of thermogenesis (25). By immunostaining, UCP1 relative fluorescence intensity was 1.50-fold greater in the 1-LGPC-treated group compared with the Ctrl group (Fig. 4D, E). Greater UCP1 expression was further validated using Western blot analysis, which revealed a 120.70% increase following 1-LGPC treatment. PGC1α, another central regulator of cellular metabolism, was also upregulated by 24.28% in the 1-LGPC treatment group. Collectively, these findings revealed a function of 1-LGPC in promoting mitochondrial respiration and thermogenesis by upregulating PGC1α and UCP1, which also prevents the accumulation of lipids.

### 1-LGPC promotes oxidative phosphorylation, ROS detoxification, and lipid remodeling

**Fig. 5:**
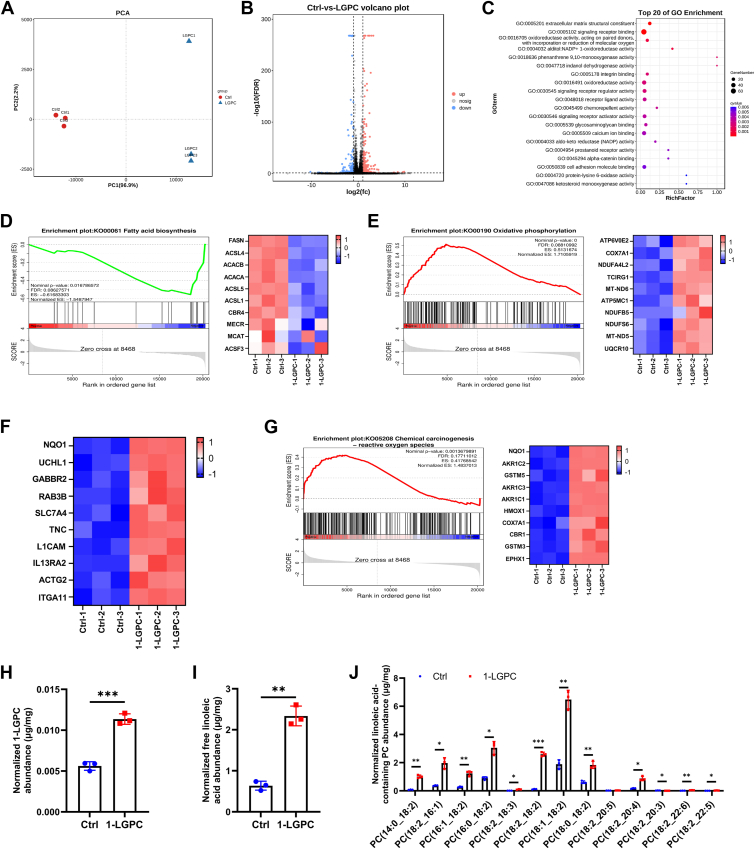
Transcriptomic and lipidomic analyses reveal induced DEGs and the metabolic fate of 1-LGPC. A: PCA score plot of transcriptomic datasets. B: Volcano plot showing DEGs. C: Top 20 molecular function categories in Gene Ontology enrichment analysis. D: GSEA of the fatty acid biosynthesis pathway and the top 10 leading-edge genes. E: GSEA of the oxidative phosphorylation pathway and the top 10 leading-edge genes. F: Top 10 ranked genes in GSEA. G: GSEA of the chemical carcinogenesis-ROS pathway and the top 10 leading-edge genes. H: Normalized 1-LGPC abundance. I: Normalized free linoleic acid abundance. J: Normalized linoleic acid-containing PC abundance. ACACA, acetyl-CoA carboxylase alpha; ACACB, acetyl-CoA carboxylase beta; ACSF3, acyl-CoA synthetase family member 3; ACSL1, acyl-CoA synthetase long chain family member 1; ACSL4, acyl-CoA synthetase long chain family member 4; ACSL5, acyl-CoA synthetase long chain family member 5; ACTG2, actin gamma 2, smooth muscle; AKR1C1, aldo-keto reductase family 1 member C1; AKR1C2, aldo-keto reductase family 1 member C2; AKR1C3, aldo-keto reductase family 1 member C3; ATP5MC1, ATP synthase membrane subunit C locus 1; ATP6V0E2, ATPase H^+^ transporting V0 subunit e2; CBR1, carbonyl reductase 1; CBR4, carbonyl reductase 4; COX7A1, cytochrome *c* oxidase subunit 7A1; COX7A1, cytochrome *c* oxidase subunit 7A1; EPHX1, epoxide hydrolase 1; GABBR2, gamma-aminobutyric acid type B receptor subunit 2; IL13RA2, interleukin 13 receptor subunit alpha; ITGA11, integrin subunit alpha 11; L1CAM, L1 cell adhesion molecule; MCAT, malonyl-CoA-acyl carrier protein transacylase; MECR, mitochondrial trans-2-enoyl-CoA reductase; MT-ND5, mitochondrially encoded NADH:ubiquinone oxidoreductase core subunit 5; MT-ND6, mitochondrially encoded NADH:ubiquinone oxidoreductase core subunit 6; NDUFA4L2, NDUFA4 mitochondrial complex associated like 2; NDUFB5, NADH:ubiquinone oxidoreductase subunit B5; NDUFS6, NADH:ubiquinone oxidoreductase subunit S6; NQO1, NAD(P)H quinone dehydrogenase 1; RAB3B, member of the RAS oncogene family; SLC7A4, solute carrier family 7 member 4; TCIRG1, T-cell immune regulator; TNC, tenascin C; UCHL1, ubiquitin C-terminal hydrolase L1; UQCR10, ubiquinol-cytochrome *c* reductase, complex III subunit X.

To better determine the role of 1-LGPC in energy metabolism and other biological processes, the transcriptomic expression profiles of 1-LGPC-treated adipocytes were compared with the Ctrl group. PCA revealed that PC1 and PC2 could explain 99.1% of the variance, indicating substantial differences in the expression profiles of 1-LGPC-treated adipocytes compared with Ctrl (Fig. 5A). Four hundred thirty-four DEGs were identified in mature adipocytes exposed to 1-LGPC, with 254 genes upregulated and 180 genes downregulated (Fig. 5B). Gene Ontology analysis revealed the association of many DEGs with oxidoreductase function and a close relationship with mitochondrial respiration (Fig. 5C), implicating mitochondrial respiration as the molecular mechanism resulting in diminished lipid accumulation following 1-LGPC treatment.

Lipid accumulation is primarily regulated by fatty acid biosynthesis, which was shown to be significantly inhibited following 1-LGPC treatment through GSEA (Fig. 5D). The transcription of genes involved in fatty acid biosynthesis, including *FASN* and acyl-CoA synthetase long-chain family member 4 (*ACSL4*), was decreased in 1-LGPC-treated adipocytes (Fig. 5D), resulting in the marked decrease in accumulation of lipids. Genes encoding ATPase H^+^ transporting V0 subunit e2 (ATP6V0E2), cytochrome c oxidase subunit 7A1 (COX7A1), and NDUFA4 mitochondrial complex associated like 2 (NDUFA4L2) were all upregulated in 1-LGPC-treated adipocytes (Fig. 5E), indicating an enhancement in the oxidative phosphorylation pathway. This upregulation is consistent with the promotion of mitochondrial respiration and thermogenesis.

Next, we aimed to identify the upstream regulators of mitochondrial respiration and thermogenesis. NAD(P)H quinone dehydrogenase 1 (*NQO1*) was upregulated by 9.35-fold and ranked first in GSEA (Fig. 5F), highlighting its role in ROS metabolism. Numerous genes involved in ROS metabolism, such as *NQO1*, aldo-keto reductase family 1 member C2, and glutathione-*S*-transferase mu 5 (*GSTM5*), were significantly upregulated (Fig. 5G). Excess ROS can deactivate components of the respiratory chain and enzymes in the tricarboxylic acid cycle (26). Studies have shown that activating ROS scavenging mechanisms and the upstream regulator NRF2 could prevent obesity and metabolic diseases (27). Therefore, the enhancement of oxidative phosphorylation and energy metabolism likely contributes to the activation of NRF2 and subsequent ROS-detoxifying pathways, offering a potential mechanism for 1-LGPC’s antiobesity effects.

In addition, lipidomic analyses were performed to determine the metabolic fates of 1-LGPC after being uptaken into adipocytes. After administration of 1-LGPC in the culture medium, the level of intracellular 1-LGPC increased 2.02-fold (Fig. 5H), indicating an effective uptake of exogenous 1-LGPC. Then, 1-LGPC could function as a regulator, hydrolyze to free 18:2, or reacylate to PC. The abundance of free 18:2 increased 3.67-fold (Fig. 5I), and the abundances of most 18:2-containing PC increased dramatically (Fig. 5J). LPC exerts multiple regulatory effects through modulating G protein-coupled and Toll-like receptors, whereas PC constitutes a primary structural element of cellular membranes (28), and free 18:2 mainly induces inflammation and adipogenesis (29). Therefore, LPC was chosen to further explore its antiobesity effects and oxidative phosphorylation activation mechanisms.

### 1-LGPC activates the KEAP1-NRF2 axis to treat obesity

**Fig. 6:**
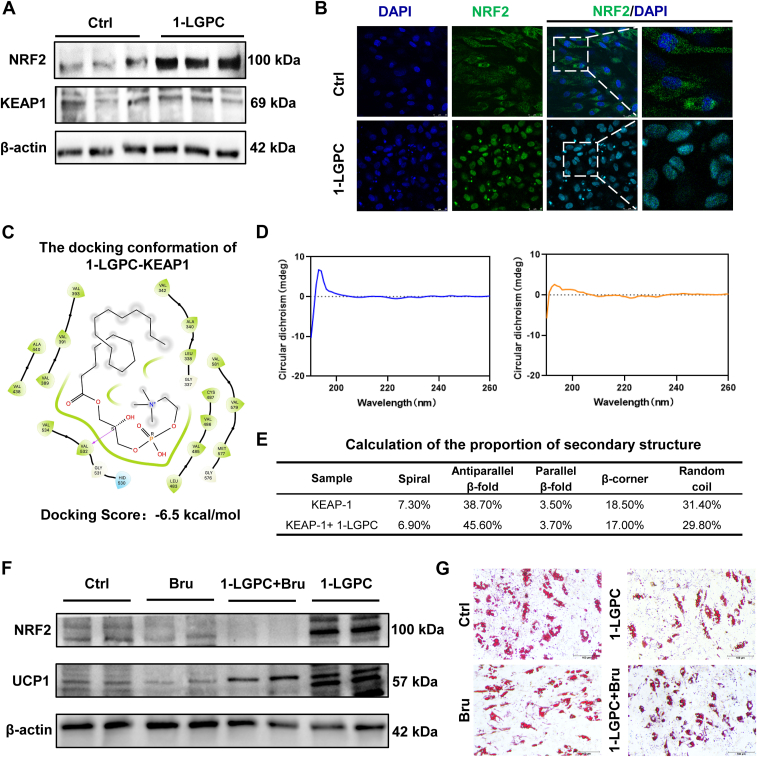
1-LGPC attenuates lipid deposition through the KEAP1-NRF2 axis. A: Immunoblot analysis of NRF2 and KEAP1 in the 1-LGPC-treated group compared with Ctrl. β-actin was used as an internal reference. B: Representative images of NRF2 immunofluorescent staining. Nuclei were counterstained with DAPI. The scale bars represent 25 μm. C: The docked conformation of 1-LGPC-KEAP1 predicted using Schrödinger software. D: Far ultraviolet CD spectrum scans of purified KEAP1 with (right) and without 1-LGPC (left). E: Calculation of the proportion of secondary structure using CDNN software. F: Immunoblot analysis of NRF2 and UCP1 in the 1-LGPC-treated group with or without Bru. G: Representative images of mature adipocytes stained by Oil Red O. Bru, brusatol; KEAP1, Kelch-like ECH associated protein 1.

NRF2 is a transcription factor that positively regulates the activation of genes encoding key protective enzymes, such as NQO1, heme oxygenase 1 (HMOX1), GST, aldo-ketoreductases, γ-glutamylcysteine ligase, thioredoxin, and thioredoxin reductase under stress conditions (19). Given that *NQO1*, *GSTM5*, *HMOX1*, and *GSTM3* were upregulated by 9.35-fold, 2.75-fold, 2.33-fold, and 1.73-fold in 1-LGPC-treated adipocytes, respectively, we investigated the expression levels of NRF2 and its upstream regulator KEAP1 by Western blotting. NRF2 was upregulated by 1.11-fold following 1-LGPC treatment, whereas the expression level of KEAP1 was not significantly different (Fig. 6A). Immunofluorescent staining revealed that NRF2 was primarily located in the cytoplasm and maintained a low expression level in Ctrl adipocytes (Fig. 6B). After 1-LGPC treatment, NRF2 expression was enhanced and showed colocalization with the nucleus, indicating translocation of NRF2 to the nucleus and activation of cytoprotective gene expression. These data suggest that 1-LGPC promotes NRF2 nuclear translocation, thereby activating its downstream protective pathways (30).

KEAP1 typically represses the nuclear accumulation and transcriptional activation activity of NRF2 to investigate the effect of 1-LGPC on the conformation of KEAP1, a docking conformation model was predicted using Schrödinger software. The results indicated that 1-LGPC interacts with KEAP1 at Val532 through hydrogen bonding and with other residues via van der Waals forces, achieving docking energy scores of −6.5 kcal/mol (Fig. 6C). The conformational change in KEAP1 induced by 1-LGPC was further demonstrated through far-ultraviolet CD spectrum scans, showing a significant decline in the positive band at 195 nm (Fig. 6D). Using CDNN software to calculate the proportion of secondary structures, it was found that the antiparallel β-sheet content of KEAP1 increased from 38.70% to 45.60% after treatment with 1-LGPC (Fig. 6E), suggesting a conformational change that might affect the KEAP1 adaptor to ubiquitin ligase.

Then, brusatol, a well-characterized NRF2 inhibitor (31), was administered to validate the involvement of the KEAP1-NRF2 axis in UCP1 activation and suppression of lipid accumulation. The pharmacological inhibition of NRF2 by brusatol administration significantly attenuated NRF2 protein levels under basal and 1-LGPC-treated conditions (Fig. 6F). Concomitantly, this intervention led to a marked downregulation of UCP1 expression. Besides, brusatol treatment effectively counteracted the 1-LGPC-induced suppression of lipid droplet accumulation in adipocytes (Fig. 6G). Collectively, the aforementioned results demonstrate that 1-LGPC activates the KEAP1-NRF2 axis to induce mitochondrial respiration and thermogenesis and therefore attenuates lipid accumulation.

## Discussion

Obesity is a multifactorial health issue that contributes to noncommunicable diseases, such as diabetes mellitus, arterial hypertension, cardiovascular disease, musculoskeletal disorders, and malignancies. LPCs are signaling molecules with significant roles in regulating cell division, inflammation, and obesity-induced low-grade inflammation (16). In our study, 1-LGPC was identified as the most abundant differential glycerophospholipid when comparing Ctrl and obese patients, with its concentration significantly declining in peripheral blood (Fig. 1A–D). It has been reported that hemodialysis patients with low LPC levels (≤254 μM) have a significantly increased risk of cardiovascular disease (32). In mice, levels of LPC 16:0, 1-LGPC, and LPC 18:1, the most abundant LPCs (80–190 μM) in plasma, reduced following the administration of an HFD diet (33). Similarly, 1-LGPC, LPC 18:1 (20–35 μM), and other LPC species with concentrations lower than 10 μM showed significant reductions in plasma concentrations in obese subjects (34). The reduction of plasma 1-LGPC was previously shown to predict impaired glucose tolerance, insulin resistance, type 2 diabetes, coronary artery disease, memory impairment, and gait speed (35). In this study, 1-LGPC levels were negatively correlated with BMI and most associated baseline characteristics, with an area under the curve value of 0.902 (0.830–0.974) in distinguishing obese patients from Ctrls (Fig. 1E). These findings implicate 1-LGPC decline in the dysregulation of obesity-associated metabolism, suggesting its potential involvement in lipid homeostasis modulation.

Then, doses of 50 μM and 100 μM were selected to investigate the role of 1-LGPC in lipid metabolism, which was shown to effectively alleviate HFD-induced lipid accumulation in zebrafish larvae (Fig. 2C-F) and human adipocytes (Fig. 3B). Genes engaged in cell differentiation and fatty acid biosynthesis were downregulated (Figs. 3C and 5D), whereas the expression level of p-HSL, a key regulator of lipolysis, was increased by 110.86% after treatment with 1-LGPC (Fig. 3D, E). The aforementioned transcriptomic changes together led to attenuated lipid accumulation, which was contrary to lysophosphatidylinositol’s role in upregulating *FASN*, *ACC,* and *PPARγ* and promoting lipid storage (36). UCP1 is a critical protein component promoting thermogenesis by permitting inefficient oxidative phosphorylation (37). Both mRNA and protein levels of UCP1 and its upstream regulator, PGC1α, were significantly increased (Figs. 3C and 4D–G) in 1-LGPC-treated adipocytes, resulting in increased energy expenditure. In line with the changes in gene expression, we observed the relative intensity and OCRs of mitochondria to be increased by 1.65-fold and 1.80–2.60-fold (Fig. 4A–C), respectively, as well as the enhancement of the oxidative phosphorylation pathway, after administration of 1-LGPC (Fig. 5E). It was reported that the biosynthesis of cardiolipins, which were essential for initiating and maintaining adipose thermogenesis, mostly occurred at the inner mitochondrial membrane (37). Unlike cardiolipins, phosphatidylethanolamine lacked documented evidence of structural interaction with UCP1; it regulates thermogenesis via modulating the electrochemical gradient across the inner mitochondrial membrane bilayer to enhance UCP1-mediated proton conductance (38). In LPCs, LPC 16:0 enhanced isoproterenol-induced UCP1-mediated leak respiration in brown adipocytes (39), and LPC 18:1 inhibited thioesterase superfamily member 1 activity to drive forward thermogenesis in brown adipose tissue (40). The aforementioned results demonstrated that 1-LGPC treats obesity by enhancing UCP1-dependent thermogenesis and mitochondrial respiration.

To uncover the regulatory mechanisms of enhanced thermogenesis mediated by 1-LGPC, transcriptomic analysis was conducted in human adipocytes. The upregulated *NQO1* ranked first in GSEA (Fig. 5F), whereas *GSTM5*, *HMOX1*, and *GSTM3*, which are also involved in the ROS detoxification, were also shown to be upregulated (Fig. 5G), potentially implicating the common upstream regulator of ROS detoxification, NRF2. NRF2 functions as a principal regulator of antioxidation and cytoprotection that functions mechanistically by binding to antioxidant response elements (AREs) and has exhibited the potential to treat obesity and type 2 diabetes (27). After 1-LGPC administration, NRF2 levels were significantly increased by 1.11-fold (Fig. 6A), and NRF2 protein was found to translocate from the cytoplasm to the nucleus (Fig. 6B). The NRF2-ECH homology (Neh) 4 and Neh5 domains comprise the NRF2 transactivation site that functions to recruit cAMP response element-binding protein-binding protein and/or receptor-associated co-activator 3 (19). This promotes high levels of expression of NRF2 target genes, including *NQO1* and *HMOX-1*, which contained ARE-binding regions in their promoters (41). Similarly, several activators of NRF2 signaling, including glucoraphanin and synthetic oleanolic triterpenoid 1-[2-cyano-3,12-dioxooleana-1,9(11)-dien-28-oyl]-imidazole, were shown to effectively attenuate HFD-induced lipid accumulation in wild-type, but not in *Nrf*2-deficient mice (42, 43), indicating the essential role of NRF2 in ameliorating obesity. It was reported that NRF2 activates UCP1 expression by directly binding DNA regions located at −3.7 and −2.0 kb upstream of the *UCP1* transcription start site, and *Nrf*2 deficiency attenuated both UCP1 levels in adipocytes and energy expenditure in obese mice (44). Meanwhile, a conserved ARE sequence was also identified in the *PGC1α* promoter, and NRF2 directly promoted *PGC1α* expression at the transcriptional level (45), leading to UCP1 activation. Beyond direct transcriptional activation, a key mediator in the NRF2 stress/oxidant response is HMOX-1, with NRF2-HMOX1-mediated mitochondrial iron regulation playing a central role in redox-stress-induced white adipose tissue browning (44, 45). Furthermore, NRF2 enhanced ROS scavenging to boost mitochondrial antioxidant capacity, promoting mitochondrial biogenesis and mitophagy to rejuvenate mitochondrial oxidative phosphorylation (26), and mitochondrial ROS modulation affected UCP1 activity (46). Collectively, NRF2 and subsequent activation of ROS detoxification pathways played a central role in UCP1-dependent thermogenesis and mitochondrial respiration.

KEAP1 binds to the DLG and ETGE motifs in the Neh2 domain of NRF2, which serves as an adaptor for a Cullin 3-based ubiquitin ligase, promoting the ubiquitination and proteasomal degradation of NRF2 under homeostatic conditions (19). We did not observe altered KEAP1 protein levels following 1-LGPC treatment (Fig. 6A). There are four cysteine residues (Cys257, Cys273, Cys288, and Cys297) within the BTB/Kelch repeat region of KEAP1, which can be oxidized by ROS or lipid oxidation products to alter the conformation of KEAP1 and subsequently release NRF2 (47). Although lipidomic analysis revealed that intracellular levels of 1-LGPC, its breakdown and reacylation products, free linoleic acid- and linoleic acid-containing PCs all increased after 1-LGPC treatment (Fig. 5H–J), neither free linoleic acid nor linoleic acid-containing PCs demonstrated significant NRF2 activation or thermogenesis effects (28, 29, 48, 49). In contrast, 1-LGPC exhibits robust physiological activity (28); therefore, 1-LGPC was selected for further investigation into its mechanisms of NRF2 activation and thermogenesis. 1-LGPC was predicted to interact with Cys487 and other residues of KEAP1, exhibiting docking energy scores of −6.5 kcal/mol (Fig. 6C), suggesting a moderate potential of KEAP1 binding. Using CD spectrum scans, the antiparallel β-fold sheet content of KEAP1 rose from 38.70% to 45.60% following 1-LGPC administration (Fig. 6D, E). Antiparallel β-fold sheets have vital functions in governing the hydrogen bonding network and conformational preferences of peptides and proteins, yet the conformational rigidity of these structures may also prevent the most optimal interactions between the side chains of the derived cluster (50, 51). Therefore, increasing the antiparallel β-fold sheet content of KEAP1 could alter the conformation of KEAP1 and prevent its interaction with Cullin 3, promoting NRF2 release, accumulation, and nuclear translocation. The rescue experiments further provided mechanistic validation that NRF2 inhibition induced downregulation of UCP1 expression and concomitant restoration of lipid accumulation (Fig. 6F, G). Previous studies have demonstrated that lysophospholipids might mediate regulatory functions through activation of G-protein-coupled receptors (17, 36). In our study, 1-LGPC exerts its antiadipogenic effects through activating the KEAP1-NRF2 axis to enhance UCP1-dependent thermogenesis and mitochondrial respiration.

Cumulatively, these data identified 1-LGPC as a major metabolite with lower abundance in the peripheral blood of obese patients. Moreover, administration of 1-LGPC resulted in attenuated lipid accumulation by enhancing UCP1-dependent thermogenesis and mitochondrial respiration. We show that 1-LGPC alters KEAP1 conformation to activate NRF2 and subsequent downstream energy expenditure pathways. Our findings elucidate the role of 1-LGPC in mitigating lipid accumulation, identifying this lysophospholipid as a promising therapeutic candidate for combating obesity.

While this study provides novel insights into the regulatory mechanisms of LPC, several limitations warrant consideration. First, the modest cohort size may constrain statistical power for definitive biomarker validation, necessitating replication in larger and demographically diverse populations. Second, despite multiple studies reporting reduced LPC levels in obese individuals, the current study lacks unambiguous structural identification of 1-LGPC to exclude potential isobaric or structurally related interferences. Meanwhile, the lack of in vivo validation through murine metabolic phenotyping, for instance, diet-induced obesity models with tissue-specific knockouts, limits physiological extrapolation. Third, confirmation of conformational modulation requires direct interaction validation and cryo-EM topology analyses. Addressing these gaps will be critical for translating these findings into therapeutic strategies.

## Data availability

The data that support the findings of this study are available from the corresponding author upon reasonable request.

## Supplemental data

This article contains supplemental data.

## Conflict of interest

The authors declare that they have no conflicts of interest with the contents of this article.
