# Supplementary material for: 1-Linoleoylglycerophosphocholine stimulates UCP1-dependent thermogenesis and mitochondrial respiration to combat obesity
Source: J Lipid Res. 2025 Sep 26;66(11):100914. doi: 10.1016/j.jlr.2025.100914 (PMC12596619; doi:10.1016/j.jlr.2025.100914)
Supplement: Supplemental data [file mmc1.pdf]

SUPPLEMENTAL INFORMATION

1-Linoleoylglycerophosphocholine stimulates UCP1-dependent thermogenesis and mitochondrial respiration to combat obesity

Rui Wang[b, †], Tianfu Zhu[b, †], Jingxian Lu[b], Mengke Cheng[c], Xingyun Wang[b], Xirong Guo[b], Shan Huang[a, *] and Jianfang Gao[a,b, *]

[a] Department of Endocrinology, Tongren Hospital, Shanghai Jiao Tong University School of Medicine, No.1111, XianXia Road, Shanghai 200336, China.

[b] Hongqiao International Institute of Medicine, Tongren Hospital, Shanghai Jiao Tong University School of Medicine, No.1111, XianXia Road, Shanghai 200336, China.

[c] Department of Pediatrics, Yixing People’s Hospital,1588 Xincheng Road, Yixing, 214200, China.

[*] Correspondence authors: Tongren Hospital, Shanghai Jiao Tong University School of Medicine, No.1111, XianXia Road, Shanghai 200336, China. *E-mail addresses:* jfgao@shsmu.edu.cn (Jianfang Gao); hs1147@126.com (Shan Huang)

[†] These authors contributed equally to this work.

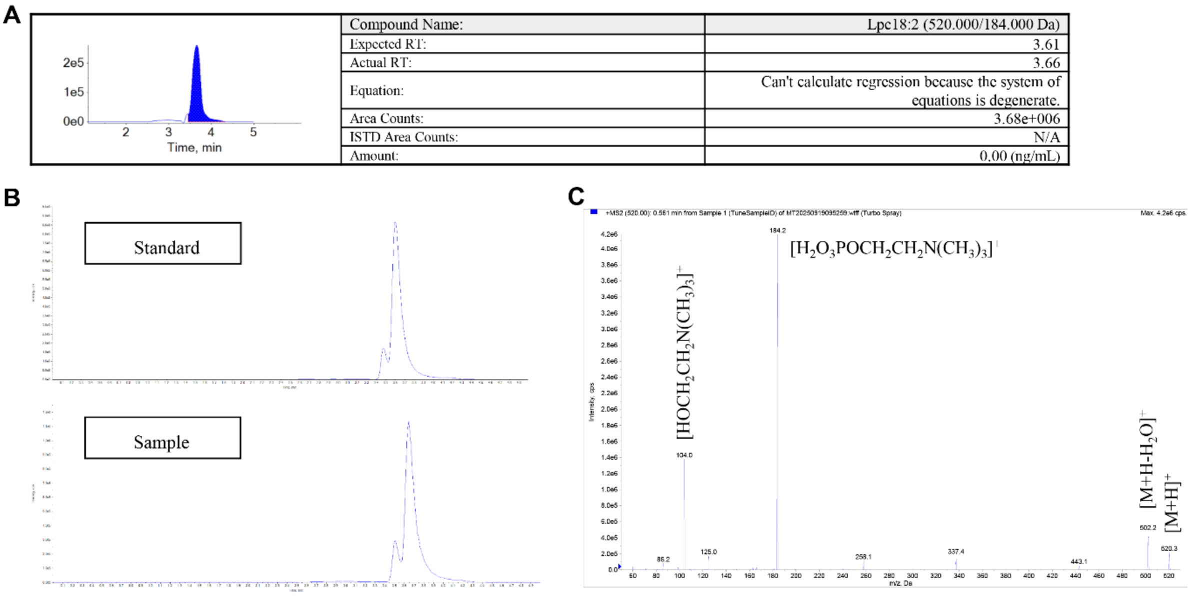

Supplemental Figure S1. The retention times and MS/MS fragmentations of standard and sample 1-linoleoylglycerophosphocholine. (A) Comparison between standard and sample 1-linoleoylglycerophosphocholine. **(B)** The retention times of standard and sample 1-linoleoylglycerophosphocholine. **(C)** Product-ion spectra of the $[M+H]^+$ ions of 1-linoleoylglycerophosphocholine.

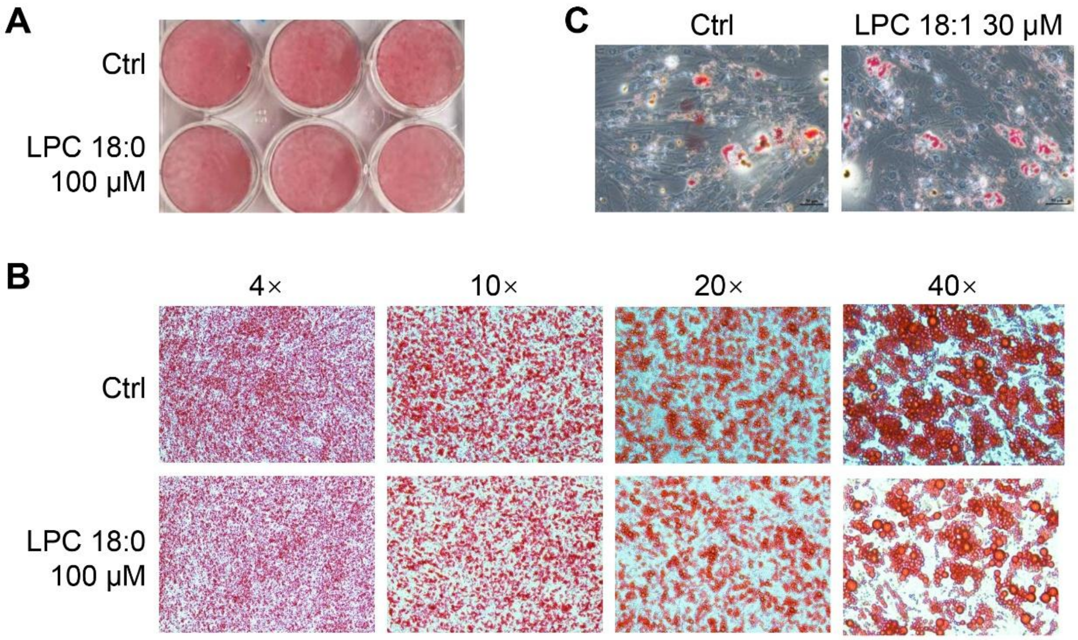

Supplemental Figure S2. Treatment of LPC 18:0 and LPC 18:1 had no significant effect on lipid accumulation in adipocytes. (A) Representative image of mature adipocytes stained by Oil Red O in six-well plates after administration of 100 μM LPC 18:0. **(B)** Representative images of mature adipocytes stained by Oil Red O after administration of 100 μM LPC 18:0. **(C)** Representative images of mature adipocytes stained by Oil Red O after administration of 30 μM LPC 18:1.
